# Association of Serum PCSK9 Levels with Antibiotic Resistance and Severity of Disease in Patients with Bacterial Infections Admitted to Intensive Care Units

**DOI:** 10.3390/jcm8101742

**Published:** 2019-10-20

**Authors:** Tannaz Jamialahmadi, Yunes Panahi, Mohamamd Amin Safarpour, Shiva Ganjali, Mahdi Chahabi, Zeljko Reiner, Saeed Solgi, Amir Vahedian-azimi, Parisa Kianpour, Maciej Banach, Amirhossein Sahebkar

**Affiliations:** 1 Halal Research Center of IRI, FDA, Tehran, Iran; JamiAT931@mums.ac.ir; 2 Department of Nutrition, Faculty of Medicine, Mashhad University of Medical Sciences, Mashhad 9177948564, Iran; 3 Pharmacotherapy Department, Faculty of Pharmacy, Baqiyatallah University of Medical Sciences, Tehran 143591647, Iran; 4 Atherosclerosis Research Center, Baqiyatallah University of Medical Sciences, Tehran, Iran; sma200111@yayhoo.com; 5 Department of Medical Biotechnology, School of Medicine, Mashhad University of Medical Sciences, Mashhad 9177948564, Iran; shivaganjali@gmail.com; 6 Department of Biochemistry, College of Basic Sciences, Shahr-e-Qods Branch, Islamic Azad University, Tehran 37515374, Iran; mahdich1404@gmail.com (M.C.); saeed.solgi2@gmail.com (S.S.); 7 University Hospital Center Zagreb, Department of Internal medicine, Kišpatićeva 12, 10000 Zagreb, Croatia; zeljko.reiner@kbc-zagreb.hr; 8 Trauma Research Center, Nursing Faculty, Baqiyatallah University of Medical Sciences, Tehran, Iran; amirvahedian63@gmail.com; 9 Clinical Pharmacy Department, Faculty of Pharmacy, Tehran University of Medical Sciences, Tehran 1417614411, Iran; pk.pioneer1@yahoo.com; 10 Department of Hypertension, WAM University Hospital in Lodz, Medical University of Lodz, Zeromskiego 113, 90549 Lodz, Poland; maciej.banach@icloud.com; 11 Polish Mother’s Memorial Hospital Research Institute (PMMHRI), 93338 Lodz, Poland; 12 Biotechnology Research Center, Pharmaceutical Technology Institute, Mashhad University of Medical Sciences, Mashhad, Iran; 13 Neurogenic Inflammation Research Center, Mashhad University of Medical Sciences, Mashhad, Iran; 14 School of Pharmacy, Mashhad University of Medical Sciences, Mashhad 9177948567, Iran

**Keywords:** PCSK9, ICU, sepsis, antibiotic resistance

## Abstract

Background: The results of several studies have suggested that infections and sepsis, either bacterial or viral, might be associated with elevated plasma proprotein convertase subtilisin/kexin type 9 (PCSK9) levels. Since there are no data on PCSK9 levels and antibiotic resistance or the severity of disease in patients with bacterial infections in intensive care units, the aim of this study was to investigate whether any such associations exist. Methods: 100 patients (46 males, mean age 67.12 ± 1.34 years) with bacterial infections who were staying in an intensive care unit (ICU) longer than 48 h but less than 7 days and who were not receiving corticosteroids were analyzed. Their serum levels of albumin, C-reactive protein, glucose, lactate, blood urea nitrogen, prothrombin (international normalized ratio), total cholesterol, triglycerides, high-density lipoprotein cholesterol, low-density lipoprotein cholesterol, serum glutamic oxaloacetic transaminase, serum glutamic pyruvic transaminase, PCSK9, and procalcitonin were measured. The severity of the patients’ condition was assessed by using the Glasgow Coma Scale (GCS), the Sequential Organ Failure Assessment (SOFA), and the Acute Physiology and Chronic Health Evaluation II (APACHE II) scales. Results: Using a hierarchical regression modeling approach, no significant association was found between PCSK9 levels and either the severity of disease (APACHE II, SOFA, and GCS) indices or resistance to antibiotics. Conclusion: The results suggest that there is no association between PCSK9 levels and resistance to antibiotics or the condition of patients hospitalized in intensive care units.

## 1. Introduction

Enzymes belonging to the proprotein convertase subtilisin/kexin (PCSKs) family are involved in the pathophysiology of different diseases, such as atherosclerosis, viral and bacterial infections, cancer, obesity, diabetes, Alzheimer’s disease, hypertension, arthritis, and even multiple trauma [1,2,3,4,5]. However, the most well-known action of one member of this family, PCSK9, in physiological conditions is connected with its capability of regulating the surface expression of low-density lipoprotein receptors (LDLRs) by targeting lysosomal degradation within hepatocytes, preventing LDLR recycling, and thereby decreasing *LDLR* concentrations and the clearance of low-density lipoproteins (LDLs) but also lipopolysaccharides (LPSes). On the basis of this, PCSK9 antibodies, more often called PCSK9 inhibitors, have been developed. They increase LDLR density on the surface of hepatocytes and thereby significantly decrease the levels of elevated LDL cholesterol in circulation [6,7]. This is particularly important in patients with very high levels of LDL cholesterol, such as those with familial hypercholesterolemia, who due to lifelong elevated LDL cholesterol levels have an increased risk for premature atherosclerotic cardiovascular disease [8,9,10].

Nevertheless, there have been a number of studies suggesting an association between increased PCSK9 levels and infection and sepsis, either bacterial or viral, which can be attributed to the modulatory effect of PCSK9 on the liver LDLR [11,12,13,14,15,16]. It seems that decreased clearance of pathogenic lipids, such as LPS from Gram-negative bacteria and lipoteichoic acid (LTA) from Gram-positive bacteria, and increased inflammatory cytokines occur due to the upregulation of PCSK9 expression, which might, at least partially, explain the important role of PCSK9 in inflammation and sepsis. LPSes and LTA are key lipid moieties of bacterial cell walls that stimulate the immune system.

It is well known that pathogenic lipids, such as endotoxins, are the trigger for the host inflammatory response in sepsis [17]. They are incorporated into lipoprotein particles such as LDL, very-low-density lipoprotein (VLDL), and HDL and are cleared from the blood by hepatocytes, which is a process mediated by LDLR [18,19]. Since the clearance of pathogenic lipids during sepsis is similar to the clearance of LDL particles, PCSK9 loss-of-function variants are associated with an increased clearance of pathogen lipids, a decreased systemic inflammatory response, and decreased one-year mortality from sepsis or in infection-related readmission after sepsis admission [20,21]. One study showed better outcomes of septic shock in patients with lower PCSK9 levels [20]. A recent study on a cohort of 10,922 patients hospitalized with infection showed that the risk of sepsis was not associated with PCSK9 genetic variations [22]. On the other hand, some studies have confirmed that PCSK9 levels are increased in septic patients, leading to decreased endotoxin clearance and increased rates of organ failure [23]. However, there have been reports indicating decreased PCSK9 concentration in sepsis and viral infections as well as PCSK9 inhibitors that have no effect on inflammation [14,24]. The results of some experimental studies have also suggested that PCSK9 inhibition provides no protection from LPS-induced mortality in mice [25]. Some experimental studies have also suggested that PCSK9 deficiency confers protection against systemic bacterial dissemination and inflammation, while PCSK9 overexpression exacerbates multiorgan pathology and proinflammatory states in early sepsis [26].

Since the results of studies on the association between serum concentrations of PCSK9 and infection and sepsis have been contradictory and since there are no data on PCSK9 levels and antibiotic resistance or the severity of disease of patients in intensive care units, the aim of this study was to investigate whether any such associations exist.

## 2. Methods

### 2.1. Patients

This cross-sectional study was performed in the general intensive care unit (ICU) of the Baqiyatallah Hospital (Tehran, Iran). This study was approved by the ethics committee of the National Institute for Medical Research Development, Tehran, Iran (code: IR.NIMAD.REC.1396.185), and written informed consent was obtained from every participant or authorized relative in case of loss of consciousness. One-hundred patients aged 18 to 80 with bacterial infections and who were staying in the ICU longer than 48 h but less than 7 days whose data with all clinical details were available were enrolled in the study (enrollment period: December 2017 to June 2018). The exclusion criteria were concomitant participation in another study and receiving corticosteroids. Patients who were discharge or died in less than 48 h or those who were included in another clinical study were excluded from this study.

### 2.2. Blood Sampling and Biochemical Measurements

Blood samples were collected from patients. Samples were centrifuged for 10 min at a speed of 1500–2000 rpm to separate the serum; to obtain plasma, blood samples (collected in tubes containing anticoagulant) were centrifuged for 20 min at a speed of 2000 rpm at 4 °C. Serum and plasma samples were kept at −70 °C until analyses. Serum levels of albumin (ALB), C-reactive protein (CRP), glucose, lactate, blood urea nitrogen (BUN), potassium (K), sodium (Na), prothrombin (PT), partial thromboplastin time (PTT), the international normalized ratio (INR), total cholesterol (TC), triglycerides (TGs), high-density lipoprotein cholesterol (HDL-C), low-density lipoprotein cholesterol (LDL-C), serum glutamic oxaloacetic transaminase (SGOT), serum glutamic pyruvic transaminase (SGPT), and the erythrocyte sedimentation rate (ESR) were measured in all patients using routine enzymatic assays and commercial kits. PCSK9 and procalcitonin (PCT) levels were measured by an ELISA according to the manufacturer’s instructions.

### 2.3. Assessment of the Severity of Disease

Level of consciousness was monitored using the GCS (Glasgow Coma Scale). A SOFA (Sequential Organ Failure Assessment) score was used to determine the status of multiple organs, such as coagulation and the cardiovascular system, respiratory system, nervous systems, liver, and kidneys. An Acute Physiology and Chronic Health Evaluation II (APACHE II) scale was used to assess the severity of the patients’ condition.

### 2.4. Statistical Analyses

Statistical analyses were performed using Statistical Package Social Sciences (SPSS) software for Microsoft Windows (SPSS 17.0, SPSS Inc., and Chicago, IL, USA) and STATA 15 (StataCorp, College Station, Texas, USA). Descriptive statistics were calculated for all patients. The chi-square test was used to statistically analyze the categorical variables. Using the Kolmogorov–Smirnov test, data were tested for a normal distribution. Variables with a normal distribution are presented as the mean and standard error, and nonparametric data are presented as the median and interquartile (IQR). A three-step hierarchical regression model was used for each outcome measure. In the first step (unadjusted model), the serum PCSK9 concentration was entered as the main predictor; in the next step (minimally adjusted model), demographic variables including age, gender, body weight, smoking, and diabetes were added to the previous model; and finally, in the third step, biochemical measures including fasting blood sugar (FBS), high-sensitivity CRP (hsCRP), lactate, BUN ALB, TG, TC, HDL-C, LDL-C, PCT, PT, PTT, SGPT, SGOT, ESR, K, and Na were further added to the aforementioned model (fully adjusted model). The change in the models at each step was assessed by partial *F*-tests, and the amount of change in the model predictability was measured by an *R*^2^ index. Additionally, to assess the moderating effect of the above-mentioned variables (in steps 2 and 3) with PCSK9, a regression model including the main and interaction effects was constructed for each outcome measure. A backward elimination strategy with a free structure was also employed (exploratory) to find the possible predictors of the outcomes. Scatter plots between the disease severity outcomes and PCSK9 were generated to assess the presence of any nonlinear association. Here, *p*-values < 0.05 were considered statistically significant.

## 3. Results

One-hundred patients were included in the study: 46 males and 54 females with a mean age of 67.12 ± 1.34. Twenty-two percent of patients had diabetes, and 39% were smokers. Their mean CRP level was 11.84 ± 0.41 mg/dL, and about 67% of patients had a CRP value above 10 mg/dL. Gram-negative bacteria were isolated from 97% of patients, and among them *Klebsiella* sp., *Pseudomonas aeruginosa*, and *Acinetobacter* were the most frequent. About 70% of patients were resistant to four antibiotics (Ceftriaxone > Ciprofloxacin > Meropenem > Amikacin). Details of the patients’ data are shown in Table 1 and Table 2.

The results of hierarchical regression modeling indicated that there was no association between PCSK9 levels and APACHE II and GCS in the first, second, and third steps of hierarchical modeling. However, in the first and second steps of hierarchical modeling, significant associations were observed between PCSK9 levels and SOFA, but after adjusting for biochemical measures for the association in the third step, the association did not remain statistically significant (*p* = 0.027) (Table 3). In the same manner, there was no association between serum PCSK9 levels and antibiotic resistance either in the unadjusted or adjusted models (Table 3).

We also used a backward elimination strategy with a free structure to exploratorily find the possible predictors of the outcomes. The results confirmed the above results, suggesting no association between PCSK9 levels and APACHE II (coefficient: −0.06; SE: 0.04; 95% confidence interval (CI): −0.01, 0.02; *p* = 0.135), SOFA (coefficient: −0.04; SE: 0.02; 95% CI: −0.09, 0.01; *p* = 0.099), and GCS (coefficient: 0.002; SE: 0.02; 95% CI: −0.05, 0.05; *p* = 0.926).

**Figure 1:**
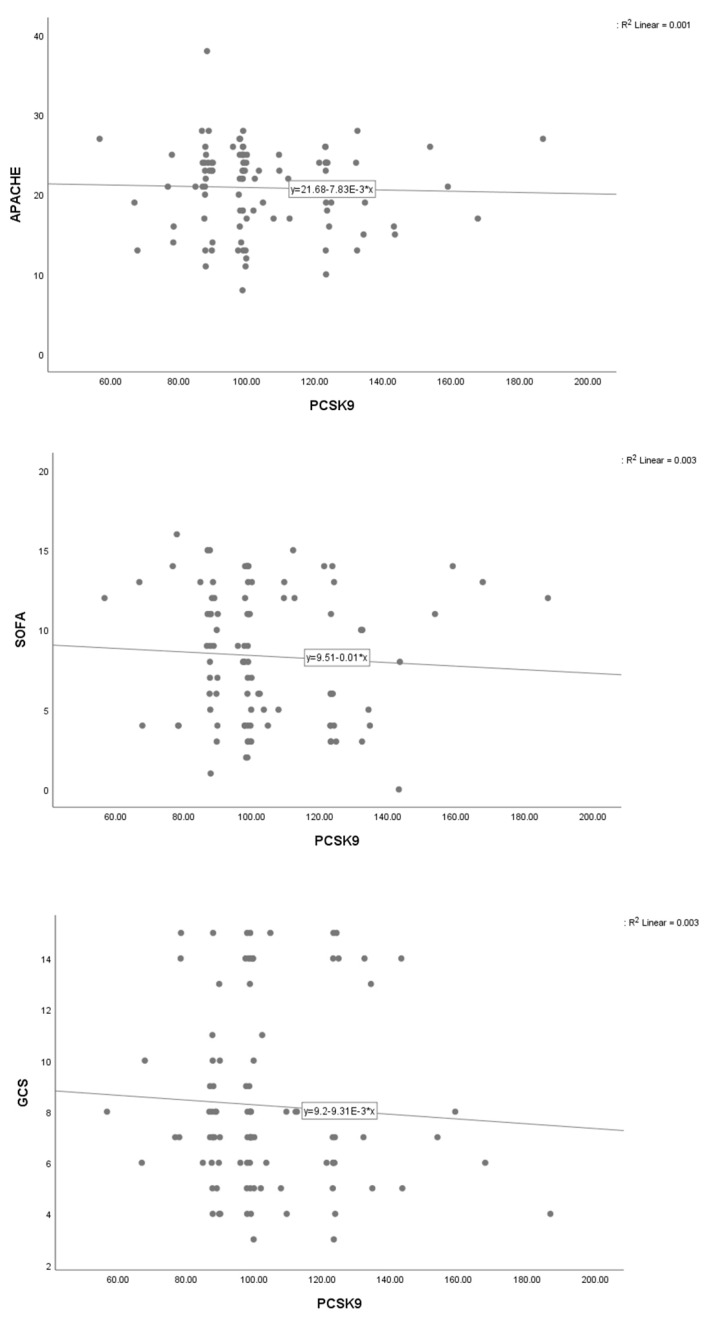
Scatter plots of association between severity of disease indices and serum PCSK9 concentrations.

We also assessed the nonlinear association (interaction) between PCSK9 levels and demographic and biochemical measures. Scatter plots of outcomes (APACHE, GCS, and SOFA) versus serum PCSK9 concentrations did not show any evidence of nonlinearity (Figure 1).

## 4. Discussion

This is the first study investigating whether there is an association between serum PCSK9 levels and resistance to antibiotics as well as the severity of disease in patients in intensive care units. The results of this study show that there was no significant association between PCSK9 levels and resistance to antibiotics or any other parameters of the severity of a patient’s condition.

It has been suggested that increased circulating PCSK9 might be associated with different bacterial and viral infections and that inhibiting the activity of circulating PCSK9 levels using PCSK9 inhibitors may be useful for the treatment of bacterial sepsis and septic shock [27]. Some authors have found that plasma PCSK9 at normal levels has no influence upon hepatocyte bacterial endotoxin clearance, but as levels rise, there is a progressive inhibition of clearance: during sepsis, PCSK9 levels are greatly increased and highly correlated with the development of subsequent multiple organ failure [23]. The presence of multiple PCSK9 loss-of-function alleles seems to decrease the risk of one-year death or infection-related readmission in sepsis survivors [21]. According to the results of some other studies, PCSK9 loss-of-function variants are not associated with an increased risk of hospitalization for a serious infection or with the odds of sepsis in patients hospitalized with serious infections [28]. Some authors have shown that plasma PCSK9 levels are elevated equally in patients with gram-positive or gram-negative bacterial infections but that particularly high levels are seen in patients with *Streptococcus pneumoniae* bacteremia; further, a reduced plasma PCSK9 response in patients with bacteremia is associated with increased mortality [29]. The results of some studies have indicated that inhibiting PCSK9 function may be an effective treatment option for both gram-positive and gram-negative sepsis [30]. According to our results, despite all these quite convincing data indicating that serious infections and sepsis might be associated with elevated plasma PCSK9 levels, it seems that there is no association between PCSK9 levels and resistance to antibiotics or the condition of patients hospitalized in intensive care units.

Our study had some limitations. The number of patients was relatively small. Therefore, the results should be considered a pilot. Another limitation might be that the patients in the ICU had different diseases, which maybe could influence PCSK9 values. An important limitation is that patients with similar conditions and diseases should be enrolled to avoid possible bias. However, this would require many more patients and a larger study, which we intend to perform in the near future. One more limitation is that the study population was relatively severe according to the high prevalence of resistant strains, low albumin level, high SOFA scores, and high incidence of bacteremia. In the larger study we are planning we will perform a stratified/subgroup analysis accordingly.

## 5. Conclusions

There is a significant amount of convincing data indicating that serious infections and sepsis might be associated with elevated plasma PCSK9 levels. Therefore, it was interesting to investigate whether there is an association between serum PCSK9 levels and resistance to antibiotics as well as the severity of disease of patients in intensive care units. Our results suggest that there is no association between PCSK9 levels and resistance to antibiotics or the condition of patients hospitalized in intensive care units.

## Figures and Tables

**Table 1:** Characteristics of the patients.

		All Samples (*n* = 100)
Age (y)		67.12 ± 1.34
Gender (%)	Female	54
	Male	46
Weight (kg)		78.82 ± 1.12
Height (cm)		162.32 ± 1.5
Smoker (%)		39
Diabetes (%)		22
FBS (mg/dL)		171.46 ± 6.43
CRP (mg/dL)		11.84 ± 0.41
Lac (mmol/L)		2 (2–4)
BUN (mg/dL)		21 (17–31.75)
ALB (g/dL)		2.9 ± 0.1
TG (mg/dL)		291.5 ± 10.14
TC (mg/dL)		179.1 ± 5.8
HDL-C (mg/dL)		34 (32–45)
LDL-C (mg/dL)		165.5 (136–192)
PCT (ng/mL)		1.6 (0.98–2.3)
PT (s)		16 (13–19)
PTT (s)		27.3 ± 0.9
INR (s)		1.4 (1.2–2)
SGPT (U/L)		23 (21–32)
SGOT (U/L)		32 (23–43)
ESR (mm/hr)		17 (15–24)
K (mEq/L)		4.31 ± 0.1
Na (mEq/L)		138.81 ± 0.82
PCSK9 (ng/mL)		98.85 (89.15–123)
APACHE II score		22 (17–24)
SOFA score		8.35 ± 0.41
GCS score		7 (6–10)

ALB: albumin; APACHE: Acute Physiology and Chronic Health Evaluation; BUN: blood urea nitrogen; CRP: C-reactive protein; ESR: erythrocyte sedimentation rate; FBS: fasting blood sugar; GCS: Glasgow Coma Scale; HDL-C: high-density lipoprotein cholesterol; INR: international normalized ratio; K: potassium; kg: kilogram; Lac: lactate; LDL-C: low-density lipoprotein cholesterol; ml: milliliter; Na: sodium; PCSK9: proprotein convertase subtilisin/kexin type 9; PCT: procalcitonin; pg: picogram; PT: prothrombin time; PTT: partial thromboplastin time; S: seconds; SGOT: serum glutamic oxaloacetic transaminase; SGPT: serum glutamic pyruvic transaminase; SOFA: Sequential Organ Failure Assessment; TC: total cholesterol; TG: triglyceride; Y: years; values are expressed as mean ± standard error (SE) or median (interquartile (IQR)).

**Table 2:** Types of isolated organisms and antibiotic resistance in the patients.

		All Samples (*n* = 100)
**Gram-negative bacteria (%)**	*Klebsiella sp.*	35
	*Pseudomonas aeruginosa*	23
	*Acinetobacter*	35
	*Pseudomonas sp.*	1
	*Escherichia coli*	3
**Gram-positive bacteria (%)**	*Staphylococcus aureus*	1
	*Enterococcus*	2
**Type of antibiotic resistance (%)**	Meropenem	82
	Vancomycin	2
	Amikacin	74
	Ciprofloxacin	83
	Ceftriaxone	86
**Antibiotic resistance (%)**	Sensitive	9
	Resistance to one antibiotic	3
	Resistance to two antibiotics	9
	Resistance to three antibiotics	10
	Resistance to four antibiotics	69

**Table 3:** Hierarchical regression modeling exploring the association between serum PCSK9 concentrations and the severity of disease indices.

Outcome Measure	Model	Coefficient	95% CI	SE	*p*−Value
APACHE II score	Model 1	−0.04	−0.12, 0.05	0.04	0.372
	Model 2	−0.05	−0.14, 0.04	0.04	0.261
	Model 3	−0.08	−0.20, 0.03	0.06	0.150
SOFA score	Model 1	−0.06	−0.11, −0.01	0.02	0.012
	Model 2	−0.07	−0.12, −0.02	0.03	0.010
	Model 3	−0.03	−0.10, 0.03	0.03	0.278
GCS score	Model 1	0.01	−0.04, 0.06	0.03	0.681
	Model 2	0.02	−0.04, 0.07	0.03	0.492
	Model 3	0.01	−0.05, 0.08	0.03	0.674
Antibiotic resistance	Model 1	1.03	1.00, 1.06	0.01	0.089
	Model 2	1.02	0.99, 1.06	0.02	0.162
	Model 3	1.03	0.96, 1.12	0.04	0.377

Model 1: unadjusted model; Model 2: minimally adjusted model for demographic variables including age, gender, body weight, smoking, and diabetes; Model 3: fully adjusted model for demographic variables plus biochemical measures including FBS, hsCRP, lactate, BUN ALB, TG, TC, HDL-C, LDL-C, PCT, PT, PTT, SGPT, SGOT, ESR, K, and Na. CI: confidence interval; SE: standard error.
